# Synthetic CT imaging for pelvic and abdominal MR‐only radiotherapy: Clinical validation on a 1.5T MR‐Linac

**DOI:** 10.1002/acm2.70571

**Published:** 2026-05-16

**Authors:** Colin Gaban, Olivier Pisaturo, Raphael Moeckli, Marc Pachoud, Sarah Ghandour

**Affiliations:** ^1^ Department of Radiotherapy Riviera‐Chablais Hospital (HRC) Rennaz Switzerland; ^2^ Institute of Radiation Physics University Hospital and Lausanne University (CHUV) Lausanne Switzerland

**Keywords:** MR‐Linac, MR‐only workflow, synthetic CT

## Abstract

**Background:**

Magnetic Resonance (MR)‐ only workflows are fundamentally limited by the absence of electron density information required for accurate photon dose calculations, necessitating supplementary CT imaging. Synthetic CT (sCT) generation algorithms offer a promising solution to eliminate this dependency. While several commercially available sCT solutions exist, their clinical scope remains highly restricted to specific MRI sequences and anatomical regions.

**Purpose:**

To evaluate two deep learning‐based sCT generation models for pelvic and abdominal anatomies using both T1 and T2‐weighted MR sequences. This study aims to validate their potential for enabling streamlined MR‐only radiotherapy workflows eliminating the need for supplementary CT imaging while reducing patient burden and workflow complexity.

**Methods:**

31 patients with multiple MRI sequences underwent sCT generation using two deep learning models (Image+, MVision AI). Geometric accuracy and Hounsfield Unit (HU) fidelity were assessed through quantitative comparison with deformed CTs (dCT) registered to MRI geometry. Dosimetric validation was performed by comparing dose distributions using γ‐index pass rates between sCT‐based plans and two reference standards: deformed CT (dCT) and clinical bulk‐density CT (bCT).

**Results:**

Mean Dice similarity coefficients demonstrated good geometric agreement: 0.73 ± 0.18 (air cavities), 0.86 ± 0.06 (adipose tissue), 0.85 ± 0.04 (soft tissue), and 0.75 ± 0.07 (bone). Dosimetric evaluation revealed averaged relative dose differences of 0.31% ± 0.98% (bCT reference) and 0.16% ± 1.13% (dCT reference) across anatomical structures. Clinical dose agreement within 2% was achieved in 98.8% of pelvic cases and 93.3% of abdominal cases.

**Conclusion:**

MRI‐based sCTs from typical T1 or T2‐weighted MR linac sequences demonstrated strong geometric and dosimetric agreement with dCT, particularly in the pelvic region, with performance appearing independent of MR sequence type. These results support the clinical viability of sCTs as an alternative to conventional CT, establishing the feasibility of comprehensive MR‐only RT workflows from simulation through treatment delivery.

## 1 INTRODUCTION

Magnetic resonance imaging (MRI) has become increasingly integrated into radiotherapy (RT) workflows due to its superior soft tissue contrast and ability to provide various contrast weightings (e.g., T1‐weighted (T1‐w) and T2‐weighted (T2‐w) sequences).^1^, ^2^, ^3^ These inherent properties significantly improve anatomical structure delineation required for precise treatment planning compared to conventional Computed Tomography (CT).^3^, ^4^ Consequently, MRI has become the standard imaging modality for various tumor sites, including prostate,^5^ brain,^6^ gynecologic regions,^7^ and rectum.^8^, ^9^

The development of hybrid magnetic resonance linear accelerators (MR‐Linacs), which integrate MRI and radiation delivery into a single platform, represents a major advancement in precision radiotherapy.^10^, ^11^ These systems enable real‐time MR‐guided radiation delivery with daily online plan adaptation,^12^ intrafraction motion tracking,^13^ and beam gating.^14^ This integrated approach allows for reduced target volume margins^15^ and decreased radiation exposure to surrounding normal tissues compared to conventional CT‐guided workflows.^16^

However, a fundamental limitation prevents full realization of MR‐only treatment planning; MRI does not provide the electron density information essential for accurate photon dose calculations.^17^ Current clinical practice addresses this limitation through hybrid approaches that combine MRI's superior imaging with CT's density information.^18^, ^19^, ^20^, ^21^ The most common implementation in MR‐Linac workflows is bulk assignment CT (bCT), where simulation CT images are deformably registered to MR geometry and average electron density values are assigned to segmented anatomical structures. In our clinical workflow, the reference treatment plan is calculated directly on the simulation MRI rather than on the CT. Therefore, a deformable registration between the simulation CT and the simulation MRI is performed to assign electron density information from CT to the MRI. This approach is applicable to both adapt‐to‐shape and adapt‐to‐position MR‐Linac workflows. While this preserves the geometric fidelity of MRI, it introduces several known limitations related to deformable registration and bulk density assignment.

First, deformable image registration between imaging sessions introduces spatial uncertainties of 1–4 mm.^22^, ^23^ potentially compromising dosimetric accuracy.^23^, ^24^, ^25^ Second, bulk‐density CT approaches require time‐intensive manual segmentation of anatomical structures to assign appropriate electron densities. Third, the requirement for both CT and MR imaging increases simulation time, workflow complexity, and patient burden. These limitations underscore the clinical need for a true MR‐only workflow^26^ that eliminates CT dependency entirely.

Synthetic CT (sCT) generation directly from MR images has emerged as the most promising solution to achieve this goal.^27^, ^28^, ^29^, ^30^, ^31^, ^32^ By eliminating CT acquisition, sCT approaches offer multiple clinical advantages: reduced ionizing radiation exposure,^31^, ^32^ decreased treatment costs,^32^ simplified workflows,^33^ and enhanced patient experience. Among various computational approaches, deep learning methods, particularly Convolutional Neural Networks (CNNs), have demonstrated superior performance.^29^, ^34^ Such sCT models consistently outperform atlas‐based and segmentation approaches, achieving improved Hounsfield Unit (HU) accuracy and image quality.^28^, ^35^, ^36^ Recent studies report dose calculation accuracies within 1% of CT‐based plans, with mean absolute errors ranging from 30–73 HU across multiple anatomical sites.^37^

Despite these promising technical results, clinical adoption of sCT remains limited.^38^ Key barriers include lack of standardized validation protocols, absence of quality assurance guidelines, and restricted generalizability of existing models, which are typically trained for single anatomical sites or specific MRI sequences.^27^, ^38^, ^39^, ^40^, ^41^ This limitation is particularly evident in the literature distribution: while 60% of sCT research focuses on pelvic applications, only 11% addresses abdominal regions, reflecting the additional challenges posed by complex anatomy, air‐tissue interfaces, and motion artifacts.^38^, ^42^

Addressing these clinical adoption barriers requires new technical implementation and validation studies that demonstrate robust performance across diverse clinical conditions. This study evaluates deep learning‐based sCT generation models (MVision AI, Helsinki, Finland) for their potential to replace simulation CT in MR‐only RT workflows. The evaluation encompasses MR imaging data acquired on the Unity 1.5T MR‐Linac system (Elekta AB, Stockholm, Sweden) using multiple MRI sequence types to reflect real‐world clinical variability. Performance is assessed for photon treatment planning in both pelvic and abdominal regions through comprehensive geometric, Hounsfield Unit accuracy, and dosimetric validation against both deformed CT (dCT) and clinical bulk‐density CT (bCT) references. This multi‐sequence, multi‐anatomical evaluation provides critical evidence for the clinical viability of streamlined MR‐only radiotherapy workflows.

## 2 MATERIALS AND METHODS

### 2.1 Patients data

This retrospective study included 31 consecutive patients treated with step‐and‐shoot intensity‐modulated radiotherapy (IMRT) on the Unity 1.5T MR‐Linac system (Elekta AB, Stockholm, Sweden) between February 2023 and January 2025. Patients were selected to ensure balanced representation across anatomical regions, MRI sequence types, and demographic characteristics. For each patient, treatment plans, simulation CT scans (simCT), simulation MR images (simMR), and corresponding structure sets were retrieved from the clinical treatment planning database.

Abdominal cohort (*n* = 15): This cohort comprised seven men and eight women with a mean age of 70.0 ± 8.5 years (range: 53–87 years). Treatment sites and corresponding MRI sequences were distributed as follows: para‐aortic lymph nodes (*n* = 2, T1‐w), pancreas (*n* = 2, T1‐w and b3DVaneXD), liver (*n* = 7, T2‐w), kidney (*n* = 1, T1‐w), bowel (*n* = 1, T1‐w), and small bowel (*n* = 2, T2‐w and b3DVaneXD). The diversity of anatomical locations and sequence types provided comprehensive evaluation of the abdominal sCT model performance. Notably, the sCT model does not require a predefined MRI sequence as input; rather, the MRI sequences used in this study correspond to those routinely selected by clinicians for each treatment site such as T1‐w, T2‐w, or balanced T1/T2 sequences.

Pelvic cohort (*n* = 16): This cohort comprised thirteen men and three women with a mean age of 70.1 ± 8.2 years (range: 46–80 years). Treatment sites included: prostate (*n* = 13, predominantly T2‐w with one T1‐w case), sacrum (*n* = 2, T1‐w and T2‐w), and rectum (*n* = 1, T2‐w). The predominance of prostate cases reflects the typical clinical distribution for pelvic MR‐guided radiotherapy.

### 2.2 Imaging

MRI Acquisition: Simulation MRI was performed on the Unity 1.5T MR‐Linac system (Elekta AB, Stockholm, Sweden) that was subsequently used for treatment delivery. This approach ensured optimal patient positioning reproducibility and compatibility with the MR treatment environment. Depending on tumor location and clinical requirements, T1‐w, T2‐w, or balanced steady‐state free precession sequences were acquired. MRI sequences included conventional T1‐w and T2‐w spin‐echo sequences, as well as specialized sequences such as b3DVaneXD (a radial acquisition designed to minimize respiratory and cardiac motion artifacts).

SimMR datasets were acquired with in‐plane resolution ranging from 0.51 to 0.94 mm (matrix sizes 480 × 480 to 800 × 800), through‐plane resolution of 1.0–1.2 mm, and 230–300 axial slices. All images were exported in DICOM format with complete geometric and intensity information preserved.

CT Acquisition: Following MRI acquisition, patients were transferred to a Big Bore CT scanner (Philips Healthcare, Cleveland, OH, USA) for simulation CT, maintaining identical positioning established during the MRI session. For abdominal treatments, a 4D‐CT was acquired using an abdominal compression belt to reduce respiratory motion; the same belt was also used during MRI simulation to ensure consistency between imaging modalities. For pelvic treatments, CT scans were acquired under free‐breathing conditions. SimCT images were acquired using standardized parameters: 1.17 mm in‐plane resolution (512 × 512 matrix), 2.0 mm slice thickness, 116–348 axial slices, and 120 kVp tube voltage. Treatment couch artifacts were subsequently removed by overriding HU values to air (−1000 HU) to eliminate dosimetric interference.

Generation of Reference Standards (bCT and dCT): In the Unity clinical workflow, the Monaco treatment planning system (TPS) (v6.2.2.0, Elekta AB, Stockholm, Sweden) only allows propagation of anatomical contours and electron density values through deformable image registration (DIR). Accordingly, bulk‐density CT (bCT) generation is performed via DIR between the simulation CT (simCT) and simulation MRI (simMR). Anatomical contours are propagated from simCT to simMR using the same deformation field, and mean electron density values derived from simCT structures are assigned to the corresponding regions on simMR. Also, in our department, the average time interval between simCT and simMR acquisitions is approximately 20 min, during which anatomical changes can occur, particularly for organs such as the bladder and rectum. To account for these changes, deformable registration is necessary to accurately fuse simCT and simMR. Non‐segmented voxels within the body contour are assigned a default electron density value of 1.0 (water‐equivalent).

For validation purposes, deformed CTs (dCT) were generated using the ANACONDA hybrid intensity‐ and contour‐based algorithm (RayStation v16.0.0.847, RaySearch, Stockholm, Sweden) with reported geometric uncertainties of 1.17 ± 0. 87 mm.^43^, ^44^ RayStation DIR was employed instead of Monaco's integrated DIR to enable comprehensive accuracy monitoring and validation. The resulting dCTs were spatially matched to simMR dimensions and resolution, providing a reference standard for geometric and dosimetric comparisons.

### 2.3 Synthetic‐CT generation

Synthetic CT images were generated from simMR using deep learning‐based models (MVision AI, Helsinki, Finland) available from the Workspace Web User interface. Two models were evaluated: Image+ Pelvis (version 1.0.0 clinically available) for pelvic anatomy and Image+ Abdomen (prototype version) for abdominal regions.

Based on manufacturer‐provided information, both models are specifically designed for photon dose calculations in external beam radiotherapy of soft tissue cancers within their respective anatomical domains. The Image+ Pelvis model was developed using 485 paired MR‐CT datasets from multiple clinical centers, while the Image+ Abdomen model was developed using approximately 250 paired datasets. Both datasets contained balanced representation of T1‐w and T2‐w sequences. Both models utilize a Conditional Convolution generator architecture adapted for medical image translation. The Image+ Abdomen model incorporates embedded bone supervision, whereby a deep learning segmentation of the complete bony anatomy, trained on 100 paired T1‐w and T2‐w samples with intensity and geometric augmentations, is provided as an auxiliary training signal to the generator network. Specific MR data augmentation techniques were applied during training. By design, the Image+ Pelvis model does not provide air cavities.

For dose calculation purposes, a standardized HU‐to‐electron density calibration curve is provided as part of the Image+ product. Comparison between our clinical calibration curve and the product‐provided curve showed differences of less than 1% in electron density values across all tissue types. Therefore, in this study, the existing clinical calibration curve from our CT scanner was retained to simplify the workflow and avoid reconfiguring of the treatment planning system.

### 2.4 sCT validation

**FIGURE 1:**
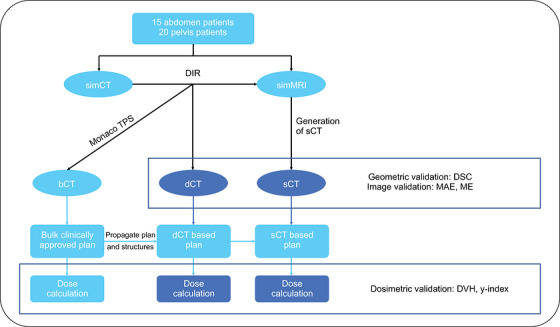
Flowchart of the validation workflow. DSC: dice similarity coefficient; ME: mean error; MAE: mean absolute error; DVH: dose volume histogram; DIR: deformable image registration; simCT: simulation CT scan; simMRI: simulation MRI image, bCT: bulk assignment CT; dCT: deformed CT; sCT: synthetic CT.

Figure 1 illustrates the dosimetric equivalence validation methodology used to assess whether simulation CT can be replaced by sCT. Our evaluation compares sCT performance against two reference standards: deformed CT (dCT) and bulk‐density CT (bCT), with the latter representing the current clinical gold standard used in our Unity MR‐Linac workflow.

### 2.5 Geometries validation

Geometric fidelity assessment between sCT and dCT was conducted within the intersection of their respective body contours to ensure fair comparison. Body contours were generated by thresholding voxels above −350 HU, and the outermost 20 mm axial slices were excluded from analysis to avoid potential artifacts from MR field‐of‐view limitations and geometric distortion effects.^3^, ^45^, ^46^

To account for methodological differences in air cavity representation, dCT regions with HU values below −500 HU that corresponded to sCT regions above −200 HU were reassigned to 0 HU, following Edmund et al.^47^ This preprocessing step eliminates artificial discrepancies arising from the sCT algorithm's inherent tendency to replace air cavities with water‐equivalent densities, ensuring that observed differences reflect genuine model performance rather than design characteristics.

Spatial agreement was quantified using Dice Similarity Coefficients (DSC) computed for four clinically relevant tissue categories defined by HU ranges: air cavities (HU < −600), adipose tissue (−150 to −20 HU), soft tissue (−20 to 120 HU), and bone (120 to 1300 HU). These thresholds were established based on electron density classifications from Lee et al.^48^ and converted to HU values using our institutional calibration curve. For pelvic cases, air cavity DSC was omitted since the Image+ Pelvis model intentionally excludes air structure generation.^49^

DSC calculations employed the standard formula:

$$DSC=\frac{2\left|V_{dCT}\cap V_{sCT}\right|}{\left|V_{dCT}\right|+\left|V_{sCT}\right|}$$

where V_dCT_ and V_sCT_ represent tissue volumes in the reference and synthetic images, respectively, with perfect agreement indicated by DSC = 1.

Hounsfield Unit accuracy was assessed through complementary error metrics:

$$\mathrm{Mean}\;\mathrm{Absolute}\;\mathrm{Error}\left(MAE\right)=\frac{1}{N}\;\sum_{i=1}^{N}\left|H{U_{dCT}}_{i}-H{U_{sCT}}_{i}\right|$$

$$\mathrm{Mean}\;\mathrm{Error}\left(ME\right)=\frac{1}{N}\;\sum_{i=1}^{N}H{U_{dCT}}_{i}-H{U_{sCT}}_{i}$$

where HU_dCTi,_ HU_sCTi_ is the HU value in the i‐th voxel of dCT and sCT respectively.

These metrics provide orthogonal information: MAE quantifies overall magnitude of HU deviations regardless of direction, while ME reveals systematic bias tendencies (positive values indicating sCT underestimation, negative values indicating overestimation).

Analysis encompassed both tissue‐specific regions and the complete body volume, with all computations implemented using custom Python algorithms (v3.13.2).

### 2.6 Dosimetric evaluation

Dosimetric validation assessed sCT performance through comparison with two reference standards: deformed CT (dCT) and bulk‐density CT (bCT), the latter serving as our institutional clinical gold standard for Unity MR‐Linac treatments.

All plans used 7 MV FFF photon beams calculated with Monaco TPS and its integrated GPU‐based Monte Carlo dose calculation engine (GPUMCD).^50^ Original clinical plans were optimized on bCT using standardized parameters: 3 mm calculation grid, dose‐to‐medium reporting, and 1% statistical uncertainty.

To ensure unbiased comparison, Monaco's direct plan transfer capability was employed, maintaining identical beam geometries, segment shapes, and monitor units across all image datasets. Each plan was systematically recalculated on dCT and sCT, isolating dosimetric differences to imaging‐related variations while eliminating confounding factors from plan re‐optimization.

Calculated dose distributions were exported to OpenTPS (v2.0.0),^51^ an open‐source research platform enabling comprehensive dosimetric analysis. Anatomical structures were consistently defined by propagating simMR contours to both dCT and sCT via rigid registration, leveraging the inherent geometric alignment between these datasets.

Structure selection reflected anatomical site‐specific clinical priorities. Abdominal evaluations included critical organs (aorta, spinal cord, liver, and bilateral kidneys) and dose‐limiting structures (bowel and stomach). Pelvic assessments encompassed genitourinary organs (bladder, rectum, colon, and penile bulb) and supporting structures (bilateral femurs).

All target volumes and organs at risk were evaluated for near‐maximum dose (D2%) and mean dose (Dmean). Target volume assessment was enhanced with coverage metrics (D95%, D98%) following ICRU Reports 50 and 62 guidelines.^52^

Spatial dose comparison employed γ‐index analysis across multiple acceptance criteria (3%/3mm, 2%/2mm, 1%/1mm, 2%/1mm) with 10% low‐dose threshold, encompassing the stringency range established in synthetic CT literature.^53^, ^54^

## 3 RESULTS

**FIGURE 2:**
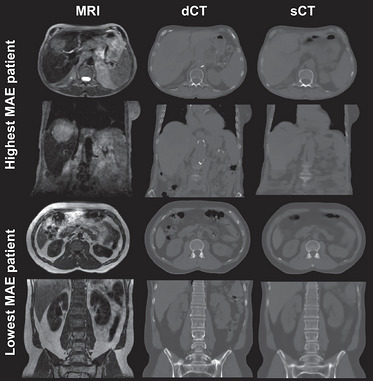
The top two first rows and the last two rows show the abdomen patient with the highest and lowest MAE values respectively. The first columns show a transverse and a coronal slice of the MR, the dCT and the sCT respectively.

**FIGURE 3:**
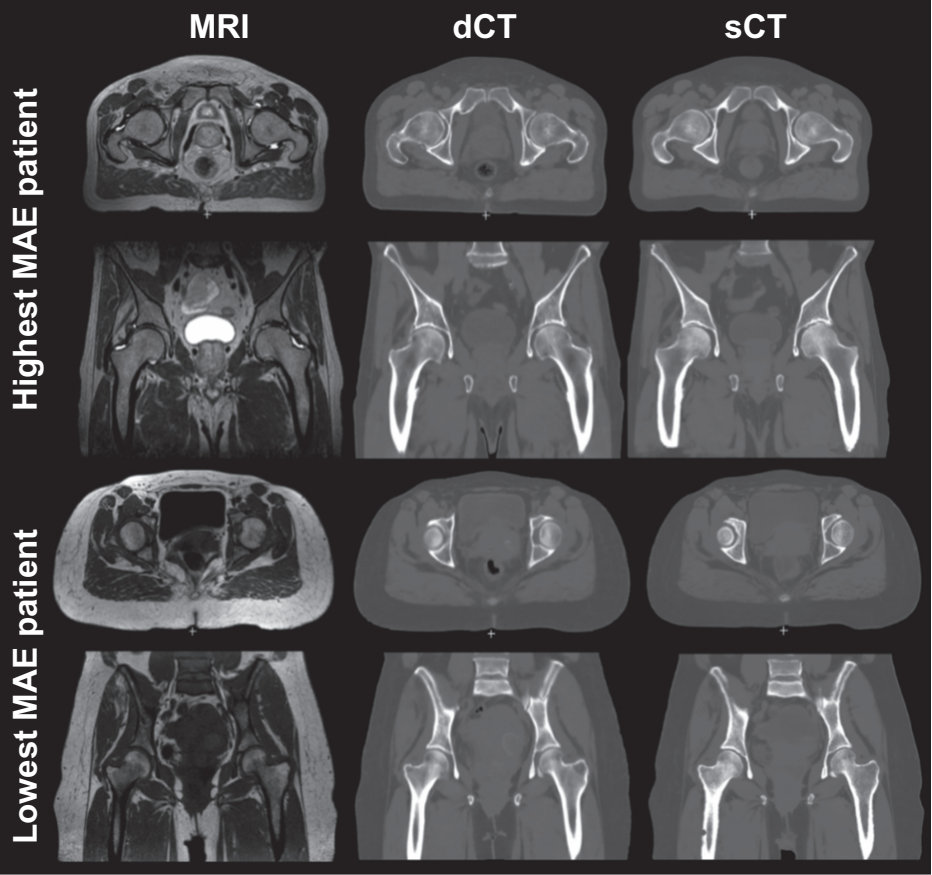
The top two first rows and the last two rows show the pelvis patient with the highest and lowest MAE values respectively. The first columns show a transverse and a coronal slice of the MR, the dCT and the sCT respectively.

The mean sCT generation time was 6.1 ± 1.1 minutes across all cases in the provided research cloud environment. Figures 2 and 3 present axial and coronal slices of simMR, dCT, and sCT images for representative cases with the best and worst performance (in terms of MAE) within the abdominal and pelvic cohorts, respectively. The sCT generation process preserved the spatial dimensions, coordinates, and frame of reference of the input MR series.

For one patient, sCT generation was unsuccessful, as the model failed to produce an output from the provided MRI data. This failure was associated with edge distortions in the input image. The corresponding simulation MRI had dimensions of [800 × 800 × 270] slices with a voxel resolution of [0.56 × 0.56 × 1.2] mm; however, the underlying cause of the failure could not be conclusively determined.

### 3.1 Geometric agreement and image validation

DSC, MAE, and ME values comparing dCT and sCT are summarized in Table 1. All DSC values demonstrated mean scores above 0.73, indicating acceptable clinical geometric agreement between image modalities.^55^, ^56^ The pelvic model achieved consistently superior performance with DSC values exceeding 0.82 across all tissue regions, while the abdominal model showed more variable performance with scores ranging between 0.67 and 0.81. The lowest DSC value (0.26) occurred for air cavities in one abdominal patient, where air pockets consisted primarily of gastric and intestinal gases rather than pulmonary air, proving more challenging for accurate sCT reproduction. This case corresponds to the worst‐performing patient illustrated in Figure 2, demonstrating discrepancies between sCT and dCT air cavity representation due to both model limitations in reconstructing air from MRI and temporal differences between MRI and CT acquisitions.

**TABLE 1:** Values for the geometric and HU‐agreement metrics between deformed computed tomography (dCT) and synthetic CT (sCT). The ME stands for Mean Error, MAE Mean Absolute Error, DSC Dice Similarity Score, *N* number of patients and HU Hounsfield units.

Cases	Air cavity HU < −600			Adipose tissue −150 < HU < −20			Soft tissue −20 < HU < 120			Bone 120 < HU < 1300			Body	
	DSC	ME	MAE	DSC	ME	MAE	DSC	ME	MAE	DSC	ME	MAE	ME	MAE
Abdomen *N* = 15	0.73 ± 0.18	−67.8 ± 118.0	130.6 ± 102.3	0.81 ± 0.08	−8.2 ± 6.5	29.4 ± 7.3	0.81 ± 0.05	10.1 ± 4.3	29.2 ± 5.5	0.67 ± 0.09	93.1 ± 25.8	136.5 ± 20.0	4.8 ± 6.8	40.8 ± 7.1
Pelvic *N* = 20	– –	– –	– –	0.90 ± 0.03	−10.8 ± 4.3	21.0 ± 4.1	0.88 ± 0.02	4.5 ± 2.3	24.5 ± 2.7	0.82 ± 0.03	9.0 ± 12.8	137.8 ± 15.7	−3.2 ± 3.6	31.8 ± 5.2
All *N* = 35	0.73 ± 0.18	−67.8 ± 118.0	130.6 ± 102.3	0.86 ± 0.06	−9.7 ± 5.3	24.6 ± 5.7	0.85 ± 0.04	6.9 ± 4.5	26.5 ± 4.7	0.75 ± 0.07	47.9 ± 44.6	137.2 ± 15.7	0.1 ± 5.7	35.8 ± 5.8

The ME and MAE mean values within the Body contour were 0.1 ± 5.7 HU and 35.8 ± 5.8 HU. The MAE and ME mean values for soft tissues were consistently lower for pelvic model (24.5 ± 2.7 HU and 4.5 ± 2.3 HU) compared to the abdomen model (29.2 ± 5.5 HU and 10.1 ± 4.3 HU). The same tendency was observed for the Adipose tissue. For bony structures, the MAE mean values were comparable across the pelvic (137.8 ± 15.7 HU) and abdominal (136.5 ± 20 HU) regions. However, the ME mean value in the Pelvis region (9.0 ± 12.8 HU) was approximately ten times lower to the abdominal region (93.1 ± 25.8 HU). In order to understand this deviation, the average ME values were calculated separately for vertebra and for ribs and results show that ME values for ribs (85 ± 27 HU) twice higher than vertebra (37 ± 23.1 HU).

**FIGURE 4:**
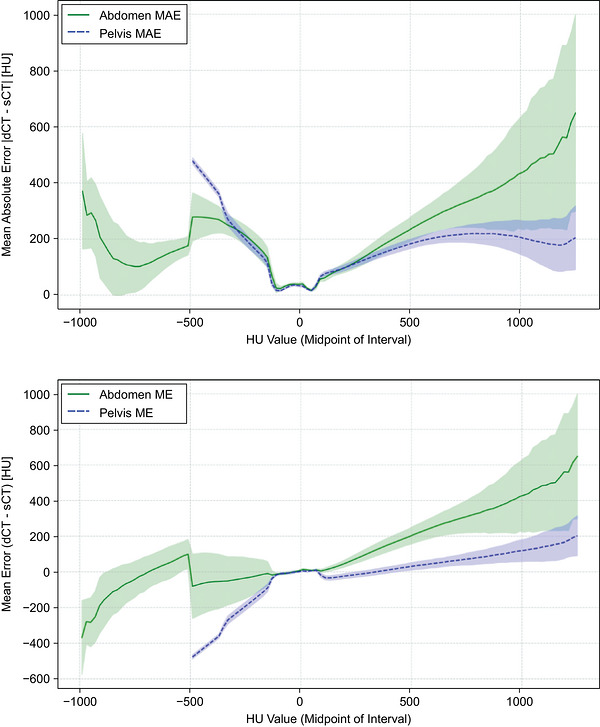
Mean absolute error (MAE) and mean error (ME) between deformed CT (dCT) and synthetic CT (sCT), computed in 20‐HU intervals, for the pelvis (dashed blue) and abdomen (solid green). Confidence bands defines one standard deviation. The drop at −500 HU shows the threshold where air pockets were set to 0 HU.

Figure 4 illustrates the MAE and ME values for abdomen (solid green) and pelvis (dashed blue) computed across 20 HU intervals using dCT as the reference standard. For the pelvic model, no values appear below −500 HU since air cavities are intentionally not reproduced by design (see Figure 3). Within the clinically relevant range of −150 to 500 HU, corresponding to most pelvic tissues, MAE remained below 200 HU and ME below 50 HU, indicating acceptable accuracy for dose calculation purposes. The abdominal model shows lower performance with significantly elevated MAE and ME values particularly within the high‐density region (above 500 HU).

**FIGURE 5:**
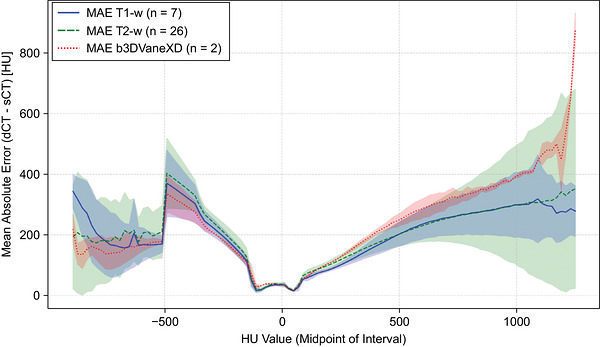
Mean absolute error (MAE) between deformed CT (dCT) and synthetic CT (sCT) in 20‐HU intervals forT1‐W (solid blue), T2‐w (dashed green) and b3DVaneXD (dotted red). The plot highlights variations in sCT quality depending on the MRI sequence used as input to the sCT generation model. Confidence bands defines one standard deviation. The drop at −500 HU shows the threshold where air pockets were set to 0 HU. T1‐w: T1‐weighted MRI, T2‐w: T2‐weighted MRI, b3D: Balanced steady‐state free precession 3D sequence.

Sequence independence analysis presented in Figure 5 demonstrates that MAE values for different MRI protocols (T1‐w, T2‐w and b3DvaneXD) remained within overlapping standard deviation ranges. This finding supports that sCT performance is independent of input MRI sequence type. The b3DVaneXD sequence, used exclusively in abdominal cases, showed comparable performance despite limited representation in the training dataset.

### 3.2 Dosimetric agreement

**FIGURE 6:**
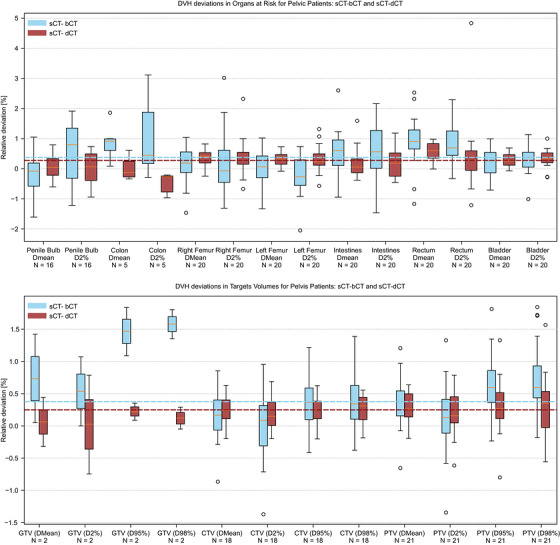
Relative dose differences between synthetic CT (sCT) and deformed CT (dCT) in blue (left box) and the sCT and bulk assignment CT (bCT) in dark red (right box). For organs‐at‐risk (OAR) and target volumes at Dose Volume Histogram (DVH) points. The dashed line represents the mean value. *N* is the number of structures evaluated.

Pelvic Region Performance: Figure 6 presents relative dose differences calculated by comparing doses obtained on sCT with those from bCT (sCT–bCT) and dCT (sCT–dCT) for various organs at risk (OARs) and target volumes (TVs) in the pelvic region.

Average relative dose differences for the sCT–bCT comparison were 0.32% (±0.82) for OARs and 0.36% (±0.53) for TVs. For the sCT–dCT comparison, discrepancies were smaller: 0.22% (±0.51) for OARs and 0.19% (±0.31) for TVs. Given the 1% statistical uncertainty of the dose calculation engine, these observed differences fall within acceptable limits.

Clinically, 98.8% of relative dose differences remained below 2.0%, with 17 of 20 patients showing all dose differences under this threshold. Dose variations ranged from −2.1% to +3.0% for OARs, except for one case where rectum D2% showed a 4.8% deviation. For this outlier, the mean organ dose was only 0.225 Gy with maximum dose of 0.926 Gy. Target volume differences were even narrower, ranging from −1.5% to +2.0%.

**FIGURE 7:**
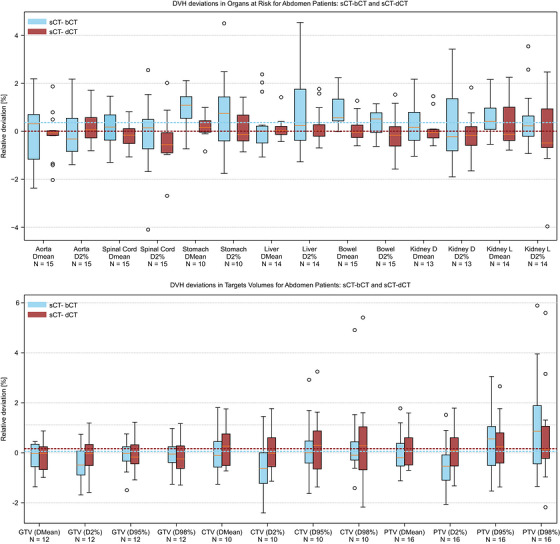
Relative dose differences between synthetic CT (sCT) and deformed CT (dCT) in blue (left box) and the sCT and bulk assignment CT (bCT) in dark red (right box). For organs‐at‐risk (OAR) and targets at Dose Volume Histogram (DVH) points. The dashed line represents the mean value. N is the number of structures evaluated.

Abdominal Region Performance: Figure 7 reports corresponding results for the abdominal region. Relative dose differences between sCT and bCT averaged 0.38% (±1.24) for OARs and 0.1% (±1.19) for TVs. The sCT–dCT comparison showed −0.02% (±0.88) for OARs and 0.23% (±1.1) for TVs.

Overall, 93.3% of relative dose differences remained below 2%, with 9 of 15 patients exhibiting all differences under this threshold. However, higher standard deviations reflect greater variability, with dose variations spanning −4.1% to +4.5% for OARs and −2.1% to +6.0% for TVs.

All cases exceeding 3.0% dose difference originated from two patients with the highest MAE values: 47 HU and 51 HU (compared to cohort average of 40 HU). Both cases involved liver tumors located near lung boundaries, regions particularly susceptible to motion artifacts during MRI acquisition.

Discrepancies were consistently more pronounced for D2% metrics compared to mean doses. Results indicate a systematic tendency for sCT to underestimate doses relative to both reference standards, as evidenced by positive mean dose differences.

Table 2 presents comprehensive γ‐index analysis results. Pass rates were consistently higher for sCT–dCT comparisons than sCT–bCT comparisons, with correspondingly lower standard deviations. The mature pelvic model generated superior dose distributions compared to the prototype abdominal model, with the latter exhibiting higher standard deviations indicating variable performance across different cases.

**TABLE 2:** γ‐index analysis performed on full 3D dose distributions of the same plan computed on a bulk assignment CT (bCT), a synthetic CT (sCT), and a CT deformed (dCT) using criteria of 3%/3 mm, 2%/2 mm, 1%/1 mm, and 2%/1 mm, with a 10% low‐dose threshold.

	Pelvis		Abdomen	
γ‐index criteria	sCT‐bCT	sCT‐dCT	sCT‐bCT	sCT‐dCT
3%/3 mm	99.87 (±0.29)	99.94 (±0.13)	98.44 (±1.4)	99.33 (±0.85)
2%/2 mm	99.48 (±0.62)	99.7 (±0.36)	96.85 (±2.3)	98.2 (±1.75)
1%/1 mm	94.87 (±2.09)	95.76 (±1.87)	89.38 (±5.86)	91.45 (±4.49)
2%/1 mm	98.94 (±0.95)	99.26 (±0.71)	95.10 (±3.39)	96.55 (±2.76)

## 4 DISCUSSION

This study evaluates synthetic CTs (sCTs) generated from MRI using two deep learning models across multiple MRI weightings (T1‐w, T2‐w, b3DVaneXD) and two anatomical regions (pelvis and abdomen) to assess model robustness under clinically realistic conditions. Performance was compared against both deformed CT (dCT) and bulk‐density CT (bCT), with the latter reflecting the clinical workflow used with the 1.5T Unity MR‐Linac. The pelvis model corresponds to a clinically released version, whereas the abdominal model was used in a validation (preclinical) version.

As shown in Figures 2 and 3, sCTs demonstrate the characteristic smoothness of deep learning models, which produce outputs resembling statistical averages of training data. While this results in loss of fine anatomical details such as small calcifications or gas cavities, it aligns with the intended purpose of sCTs: estimating electron density for dose calculations rather than diagnostic imaging. The pelvic model demonstrates superior geometric fidelity with DSC values exceeding 0.82 HU across tissue regions, while the abdominal model, trained on approximately half the dataset size, performs less reliably within bony structures particularly ribs structures which remained challenging to reproduce and were frequently underestimated in sCTs. This limitation reflects the inherent challenge of visualizing bones on MRI and the region's susceptibility to motion artifacts, leading to partial or complete omission in generated images.

When ME values are small in comparison to MAE values, this indicates mutual error compensation along photon beam paths, potentially reducing dosimetric impact. For the pelvic model, ME values remain significantly below MAE between −150 HU and 500 HU, suggesting minimal systematic bias in clinically relevant tissue ranges. Conversely, the abdominal model shows marked underestimation of bone HU values above 500 HU, where ME approaches MAE, indicating clear systematic bias. Figure 5 demonstrates that MAE values for T1‐w and T2‐w sequences fall within overlapping standard deviation ranges, indicating performance independence from MRI sequence type. The b3DVaneXD sequence showed slightly elevated MAE for HU values above 100, potentially attributable to limited representation in the training dataset.

The geometric results for pelvis fall within the upper range of literature values,^28^, ^37^ confirming model maturity. Abdominal performance, while lower (mean DSC for bone: 0.67 ± 0.09 versus published values up to 0.81^37^), remains promising for a prototype version. Dose differences below 2% were achieved in 98.8% of pelvic cases and 93.3% of abdominal cases, meeting generally accepted clinical tolerances. The systematic tendency toward dose underestimation by sCT reflects the observed HU underestimation patterns. Gamma‐index pass rates align with literature values, with the pelvic model achieving 94.8% (±2.09) at 1%/1mm and 99.4% (±0.62) at 2%/2mm criteria.^37^

Dose discrepancies correlate with MAE values,^57^ showing explaining higher relative errors in abdominal regions. Cases exceeding 3% dose differences originated from patients with highest MAE values (46–52 HU), typically involving liver tumors near lung boundaries where motion artifacts are pronounced. While air cavities and poorly reconstructed ribs showed minimal dosimetric impact for IMRT and VMAT techniques,^58^, ^59^ robust quality assurance protocols remain essential before clinical implementation.

This study demonstrates the feasibility of using the Unity MR‐Linac for radiotherapy workflows in the pelvic and abdominal regions. The results support the use of the Unity MR‐Linac as a standalone simulation platform, enabling integrated workflows in which simulation and treatment can be performed without patient repositioning. This facilitates fully MR‐based adaptive radiotherapy, where real‐time sCT generation enables dose calculation using MRI alone, eliminating the need for separate CT imaging sessions. Nevertheless, limitations remain, including image uniformity and motion‐related artifacts associated with longer MRI acquisition times. In addition, the platform‐specific validation performed on the Unity system may limit direct generalizability to other MR‐Linac platforms.

In our clinical practice, patients treated for bone targets in the abdomen are currently excluded. This decision is not driven by dosimetric concerns, as our results show high agreement between dose distributions, with gamma‐index pass rates of 2%/2 mm. Instead, the exclusion is primarily based on geometric considerations: the abdominal model tends to underestimate bone volumes, which could potentially result in target underdosage. For the moment, we also exclude patients in whom air cavities are located very close to or overlapping with the PTV.

These patient selection criteria highlight current limitations in the abdominal region and provide context for the scope of this study. Another limitation is that this work evaluates a single commercial sCT generation solution, namely the MVision AI software, using two deep learning models applied within the Unity MR‐Linac workflow. While the results demonstrate the feasibility of this approach in our clinical setting, future work could compare these findings with alternative sCT generation solutions evaluated under the same workflow. Such comparisons would be particularly valuable for the abdominal region, where available models are less developed and additional validation is required.

## 5 CONCLUSION

This study validates deep learning‐based sCT generation models for MR‐only radiotherapy on the Unity MR‐Linac, demonstrating clinically acceptable geometric and dosimetric accuracy across T1‐weighted and T2‐weighted sequences in pelvic and abdominal regions.

The pelvis model achieved robust performance with DSC values >0.82 and dose agreement within 2% in 98.8% of cases, while the prototype abdominal model met 2% dose criteria in 93.3% of cases. Performance remained consistent across both sequence types, confirming model robustness for diverse clinical protocols.

These results support eliminating supplementary CT imaging, enabling streamlined, radiation‐free simulation workflows that enhance the Unity system as a standalone platform for both simulation and adaptive treatment. The validation establishes a foundation for fully integrated MRI‐guided radiotherapy where real‐time sCT generation could support on‐the‐fly dose calculations for dynamic plan adaptation.

## AUTHOR CONTRIBUTIONS

**Colin Gaban**: Writing; data acquisition; analysis; interpretation; investigation; methodology. **Olivier Pisaturo**: Review and editing; methodology; conceptualization. **Raphael Moeckli**: Review and editing; methodology; conceptualization; supervision; resources; project administration. **Marc Pachoud**: Review and editing; methodology; conceptualization; supervision; resources; project administration. **Sarah Ghandour**: Review and editing; methodology; conceptualization; supervision; resources; project administration.

## CONFLICT OF INTEREST STATEMENT

The authors declare no conflicts of interest.
